# An Unusual Presentation of Nicolau Syndrome in the Upper Limb: A Case Report from Northern Ecuadorian Amazonia

**DOI:** 10.3390/jcm15051756

**Published:** 2026-02-26

**Authors:** Elías David Guamán-Charco, Cesar Espinoza, María Belén Vélez-Altamirano, José Govea, Willam Valdez, Guillermo Prieto-Marín, Jorge Vasconez-Gonzalez, Juan S. Izquierdo-Condoy, Esteban Ortiz-Prado

**Affiliations:** 1 Emergency Department, Marco Vinicio Iza General Hospital, Nueva Loja 210202, SC, Ecuador; 2 General Surgery Department, Marco Vinicio Iza General Hospital, Nueva Loja 210202, SC, Ecuador; jose.govea@hmvi.gob.ec; 3 Traumatology Department, Marco Vinicio Iza General Hospital, Nueva Loja 210202, SC, Ecuador; 4 One Health Research Group, Faculty of Medicine, Universidad de las Américas, Calle de los Colimes y Avenida de los Granados, Quito 170137, Ecuadorjuan.izquierdo.condoy@udla.edu.ec (J.S.I.-C.)

**Keywords:** Nicolau syndrome, embolia cutis medicamentosa, livedoid dermatitis, self medication, drug administration

## Abstract

Nicolau syndrome, also known as embolia cutis medicamentosa, is a rare iatrogenic reaction that may occur following parenteral drug administration, including inadvertent intra-arterial or periarterial injection. Its pathophysiology remains poorly understood; however, several mechanisms have been proposed, including vasospasm, embolization, cytotoxic inflammation, and secondary tissue necrosis. We report the case of a 22-year-old transgender woman who received intravenous benzathine penicillin in the left arm without a medical prescription following a reactive syphilis screening performed outside a formal healthcare setting. She subsequently developed severe pain, livedoid dermatitis, pallor, distal cyanosis, and blister formation. Radial and brachial pulses remained palpable, and Doppler ultrasonography revealed no evidence of arterial or venous thrombosis. Medical management included daily wound care, anticoagulation, corticosteroids, peripheral vasodilators, antibiotic therapy, and analgesia. The patient was hospitalized for nine days, with partial clinical improvement. However, persistent distal ischemic changes involving the second through fifth fingers raised concern for evolving necrosis and potential amputation. After counseling regarding these risks, the patient requested voluntary discharge. This case underscores the importance of safe medication administration and appropriate injection practices, particularly in low-resource settings. It also highlights the need for improved training of healthcare personnel to ensure early recognition and prompt management of Nicolau syndrome, as well as strengthened patient education to discourage self-medication and promote timely care by qualified healthcare professionals.

## 1. Introduction

Nicolau syndrome, also known as embolia cutis medicamentosa or livedoid dermatitis, was first described in 1924 by Freudenthal and later in 1925 by Nicolau, who histologically identified bismuth salts in peripheral arteries following their administration for the treatment of syphilis at that time [1,2,3]. This syndrome is considered iatrogenic in nature and has been reported more frequently in children under three years of age [1,2,4,5,6].

Although the pathophysiology of Nicolau syndrome remains poorly understood, Taştekin et al. have described several mechanisms that may contribute to its development. The process may begin with stimulation of the sympathetic nervous system triggered by the pain associated with intra-arterial or periarterial drug injection, leading to vasospasm and subsequent ischemia [7]. Stimulation of sympathetic nerves results in the release of norepinephrine from sympathetic varicosities. This neurotransmitter diffuses and binds to postjunctional α-adrenergic receptors, inducing contraction of vascular smooth muscle and, consequently, vasoconstriction. In addition, sympathetic neurons release neuropeptide Y and adenosine triphosphate, which further contribute to the overall vasoconstrictive response [8]. Nonsteroidal anti-inflammatory drugs have also been implicated, as they suppress prostaglandin synthesis, potentially contributing to ischemic injury through vasospasm mediated by inhibition of the cyclooxygenase pathway [7]. Moreover, intra-arterial drug injections may result in embolic occlusion. Ischemic necrosis may further progress due to vascular damage associated with cytotoxic inflammatory reactions to the injected agents. Finally, lipophilic drugs may enter the bloodstream in a manner similar to fat embolism, resulting in mechanical vascular occlusion [7].

We present the first reported case of Nicolau syndrome in the Ecuadorian Amazon, highlighting its clinical presentation, diagnostic challenges, therapeutic approach, and outcome in a setting with limited access to healthcare. This report aims to increase awareness among healthcare professionals and to contribute to the limited literature on this rare iatrogenic condition.

## 2. Case Report

A 22-year-old transgender woman presented to the emergency department of Marco Vinicio Iza General Hospital approximately 12 h after undergoing a first-level screening for sexually transmitted infections (STIs), specifically for syphilis, which yielded a reactive result. The patient reported that she had asked a friend to administer intravenous benzathine penicillin into her left arm. The injection was administered in the proximal region of the forearm, anterior to the elbow. The administered dose was unknown, and it could not be determined whether aspiration had been performed prior to injection. Notably, the medication was administered by an individual who was not a healthcare professional and therefore lacked formal training in medication administration. Following the injection, the patient developed progressive distal pallor of the left hand, worsening pain, and purplish discoloration. She initially sought care at a private clinic, where she received unspecified treatment. Due to persistent symptoms, she subsequently presented to our institution for further evaluation.

**Figure 1:**
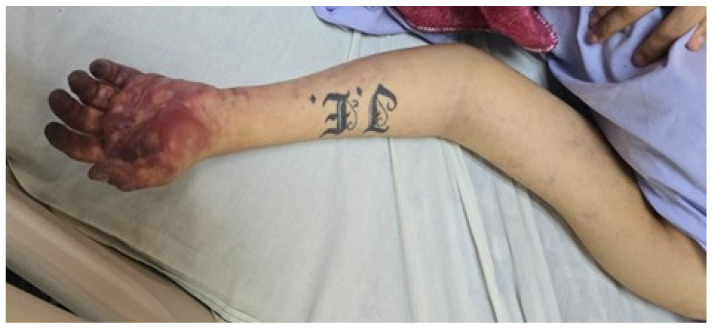
Admission to the emergency department. Blisters were observed on the palmar region of the hand, with distal cyanosis of fingers 1 through 5. Additionally, livedoid dermatitis was noted on the forearm and upper arm. Brachial and radial pulses were present.

**Figure 2:**
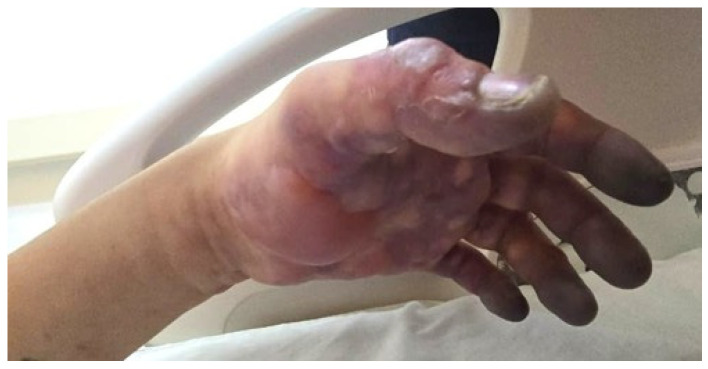
Admission to the emergency department. Blisters were observed on the dorsal region of the first thumb. Brachial and radial pulses were present.

On admission, the patient was assessed as follows:

- Patent airway.
- Preserved vesicular breath sounds, oxygen saturation of 96% on room air, and a respiratory rate of 18 breaths per minute.
- Regular heart sounds, heart rate of 73 beats per minute, blood pressure of 130/90 mmHg, with bilateral radial pulses present.
- Conscious and oriented to person, place, and time; Glasgow Coma Scale score of 15/15.
- Examination of the left upper limb revealed distal cyanosis and pallor of the fingers, blisters on the palmar surface, and livedoid dermatitis involving the forearm and upper limb. The limb was cold and moderately tender to palpation, with marked edema (+++/++++). Radial and brachial pulses were palpable. The patient’s weight was 60 kg (Figure 1 and Figure 2).

Laboratory evaluation included a complete blood count, blood chemistry, and coagulation tests (Table 1). Doppler ultrasonography of the left upper limb revealed no evidence of arterial or venous thrombosis. The patient was evaluated by multiple specialties, including traumatology and vascular surgery, which recommended intensive medical management consisting of analgesia (ketorolac 30 mg IV every 8 h), anticoagulation (enoxaparin 60 mg SC every 12 h), corticosteroid therapy (dexamethasone 8 mg IV daily), pentoxifylline 400 mg orally every 12 h, and daily wound care. Additionally, the internal medicine team recommended antibiotic therapy with ceftriaxone 1 g IV every 12 h for five days.

**Figure 3:**
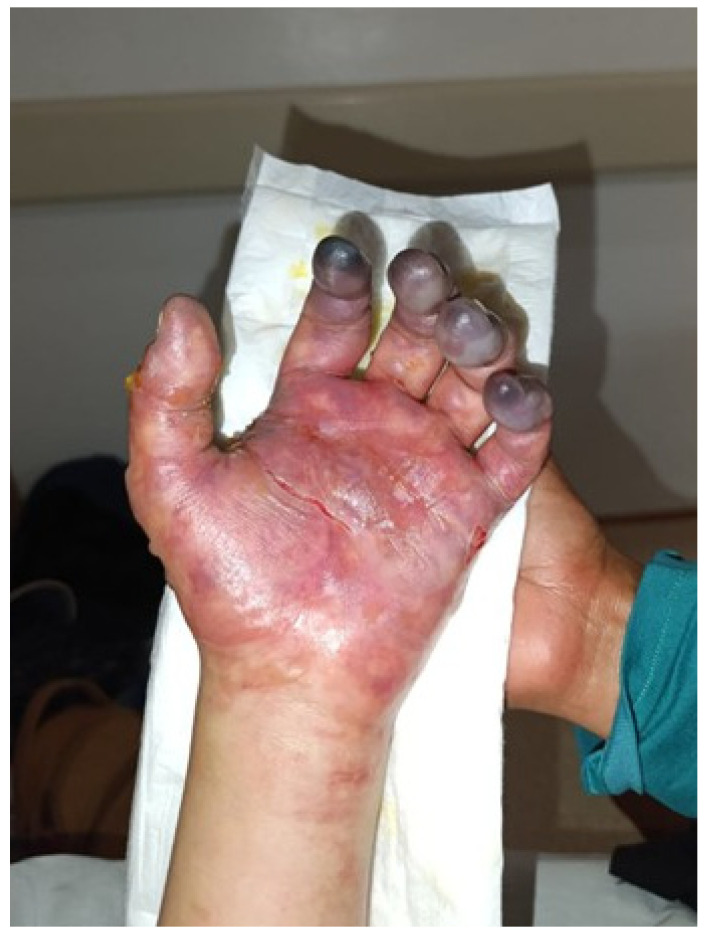
Day 9. A decrease in blisters was observed; however, persistent cyanosis remained in fingers 2 through 5.

The patient remained hospitalized for nine days and was hemodynamically stable throughout her stay. Follow-up examination of the left upper limb demonstrated a reduction in blistering and improvement in cyanosis; however, distal cyanosis persisted in the second through fifth fingers (Figure 3). Continued inpatient management was recommended. After being informed of the high risk of amputation, the patient requested voluntary discharge.

## 3. Discussion

Nicolau syndrome is a rare manifestation of drug administration (Table 2). This manuscript describes an unusual case of Nicolau syndrome secondary to inappropriate administration of benzathine penicillin. The injection was performed not by trained healthcare personnel but by a friend of the patient; consequently, the administered dose was unknown. The patient was a young transgender woman residing in the Ecuadorian Amazon, highlighting significant gaps in access to safe and timely healthcare among vulnerable populations [9,10]. In an analysis of 150 articles involving patients with Nicolau syndrome, the most frequent route of administration was intramuscular (79.05%), followed by subcutaneous (11.43%) and intravenous (3.81%) [11].

A wide range of medications has been implicated in this rare syndrome, including nonsteroidal anti-inflammatory drugs (diclofenac, piroxicam, ketoprofen, ibuprofen, and phenylbutazone); antibiotics (penicillin derivatives, tetracycline, sulfapyridine, streptomycin, gentamicin, and cephalosporins); corticosteroids (betamethasone, dexamethasone, triamcinolone, paramethasone, cortivazol, and hydrocortisone); antipsychotic and antiepileptic agents (phenobarbital and chlorpromazine); vaccines (diphtheria, tetanus, and pertussis); antihistamines (diphenhydramine and hydroxyzine); local anesthetics (lidocaine); and other agents (interferon alpha, cyanocobalamin, bismuth, and vitamin K) [6,28]. In addition, Nicolau syndrome has been reported following the use of calcium hydroxide in dental procedures [29] and after the administration of sclerosing agents such as glatiramer acetate [30]. Mojarrad et al. reported that diclofenac and benzathine penicillin are the drugs most frequently associated with this syndrome [11].

The diagnosis of Nicolau syndrome is primarily clinical, as no standardized diagnostic criteria have been established [31,32]. Accordingly, a detailed medical history, including precise information regarding drug administration, is essential. Mojarrad et al. described three clinical phases of Nicolau syndrome. The initial phase (lasting 1–3 days) is characterized by intense pain, hypersensitivity, and color changes at the injection site, along with paresthesia, erythema, livedoid-type dermatitis, syncope in some cases, edema, fever, and spasms at the affected site. The acute phase (lasting 5–10 days) is marked by discoloration and violaceous livedoid lesions at the injection site, soft tissue infection, vomiting, urosepsis, and absence of pulses. Finally, the necrotic phase (lasting from five days to two weeks) presents with necrotic, crusted, and indurated plaques at the injection site or along the affected limb, often accompanied by secondary infection [11]. It is important to note that immunosuppression, diabetes, age over 50 years, malnutrition, and obesity may increase susceptibility to Nicolau syndrome [22,33,34].

The clinical presentation may overlap with other conditions, and the main differential diagnoses include infectious entities such as cellulitis and necrotizing fasciitis, as well as vasculitis, cutaneous cholesterol embolism, and compartment syndrome [18]. Timely recognition of the characteristic signs and symptoms across the different clinical phases is therefore essential for appropriate management [11]. While imaging exams such as ultrasound, computed tomography (CT), or magnetic resonance imaging (MRI) may help define the extent of tissue involvement, these modalities are not considered first-line diagnostic tools [2,35]. One case report suggested that sequential infrared thermography may be useful for assessing treatment response during the acute phase and for delineating the extent of necrosis [32].

To date, no standardized treatment has been established, and management may vary according to the clinical phase [11]. Commonly reported interventions include analgesia, antibiotics, peripheral vasodilators, corticosteroids, and anticoagulants. In more severe cases, debridement, thrombolytic therapy, hyperbaric oxygen therapy, and plastic or reconstructive surgery may be required. Importantly, local cold application can exacerbate necrosis by inducing vasoconstriction and worsening ischemia [2].

Evidence suggests that anticoagulants and corticosteroids may help reduce vascular occlusion and inflammation during the acute phase [32]. Pentoxifylline has also been reported to relieve vasospasm and accelerate recovery from necrosis [32]. By inhibiting phosphodiesterase III, pentoxifylline reduces blood viscosity and improves microcirculatory blood flow [36]. Recently, a case report described the use of mucopolysaccharide polysulfate in a 34-year-old female patient who developed Nicolau syndrome following the injection of glatiramer acetate in the periumbilical region. This topical agent is chemically similar to heparin and improves microcirculation and perivascular tissue nutrition while inhibiting microthrombus formation [18].

Although compartment syndrome is uncommon, it has been reported as a complication in some cases [37]. One case report described a 15-month-old female patient who received benzathine penicillin in the gluteal region and subsequently developed compartment syndrome in the left calf. During the course of the illness, the tips of the toes became gangrenous, ultimately requiring calf fasciotomy and amputation [38]. Furthermore, when necrosis develops, local wound care combined with repeated surgical debridement is necessary in most cases [37]. According to a study documenting 150 cases of Nicolau syndrome, therapeutic measures included debridement in 12% of cases, fasciotomy in 4%, and amputation in 2% [11].

A recovery period of 6–8 weeks has been described [28], although recovery within 4 weeks has also been reported [39]. A distinctive feature of the present case is the unusual anatomical location. Benzathine penicillin is typically administered intramuscularly in the gluteal region; however, in this instance, self-medication resulted in the drug being administered to the left arm. To our knowledge, this represents the first case report of benzathine penicillin-associated Nicolau syndrome affecting the upper limb in the northern Ecuadorian Amazon.

## 4. Conclusions

Nicolau syndrome is a complication that is potentially preventable if appropriate injection techniques are used and autoinjectors (when indicated) are handled correctly. Health personnel should be adequately trained to ensure safe administration practices and to promptly recognize this adverse event when it occurs. In addition, this case underscores the need for patient education regarding the risks of self-medication and the importance of seeking timely, professional medical care for diagnosis and treatment.

## Figures and Tables

**Table 1:** Laboratory results at admission, Day 6, and Day 9, including complete blood count, blood chemistry, serologic tests, and coagulation parameters.

Parameter	Admission	Day 6	Day 9	Reference Range
Complete Blood Count				
Leukocytes (×10^3^/uL)	16.13	11.36		5.00–10.00
Neutrophils (%)	82.9	62.5		46–62
Lymphocytes (%)	11.3	28.6		28–44
Eosinophils (%)	0.1	0.2		1–6
Platelets (×10^3^/uL)	397	338		150–450
Red Blood Cells (×10^3^/uL)	4.57	4.05		4.30–5.70
Hemoglobin (g/dL)	13.7	12		13.20–17.80
Blood Chemistry				
Glucose	123.9			
Urea (mg/dL)	16.5		30.1	10–48.5
Creatinine (mg/dL)	0.6		0.9	0.80–1.30
VDRL	6.16			
HIV 1–2	Non-reactive			
Coagulation Times				
Prothrombin Time (s)	10.4			9.5–14
Partial Thromboplastin Time (s)	28.7			22–40
INR	0.81			
D-Dimer	Without supplies			<1.0

Abbreviations: VDRL, venereal disease research laboratory; HIV, human immunodeficiency virus; INR, international normalized ratio.

**Table 2:** Reported Cases of Nicolau Syndrome Published Between 2017 and 2026: Patient Characteristics, Drugs Involved, Clinical Features, Management, and Outcomes.

Author	Sex/Age	Drug Involved	Route of Administration	Anatomical Site	Main Clinical Features	Management	Outcome
Navrazhina et al. (2017) [12]	22-month-old girl	Lidocaine	Not specified.	Lumbosacral area	Retiform purpuric patch on the lumbosacral area	Mupirocin ointment followed by white petrolatum impregnated gauze and non-adherent dressings. Debridement of necrotic areas and a split-thickness skin graft	The wound in the lumbosacral area healed with mild hypertrophy.
Kimbrough & Newsome (2017) [13]	43-year-old woman	Glatiramer acetate	Subcutaneous route	Anterolateral aspect of the left thigh	Necrotic lesion with granulation tissue but without signs of infection or large-vessel thrombosis.	Surgical debridement followed by conservative wound care	The lesion improved
Kimbrough & Newsome (2017) [13]	34-year-old woman	Glatiramer acetate	Subcutaneous route	Left anterior thigh	Pain, erythematous and ulcerated lesion	The lesion was managed conservatively with topical wound care and dressing changes. After two weeks, an eschar had formed, and the wound was debrided	The lesion healed
Ozlu et al. (2017) [14]	81-year-old woman	Etofenamate	Intramuscular	Left gluteal region	Painful necrotic ulcer in the left gluteal region	Local wound care with saline solution once daily, topical 2% mupirocin twice daily, and surgical debridement	The ulcer healed, leaving an atrophic scar.
Gal et al. (2020) [15]	52-year-old woman	Demerol and Phenergan	Intramuscular	Left anterolateral thigh	Pain, violaceous patch at the site of the injection; lower limb swelling, but no paresthesia	Pain management included narcotics, nonsteroidal anti-inflammatory drugs, muscle relaxants, and Lyrica, along with antibiotics.	The syndrome resolved without requiring surgical debridement.
Raju et al. (2020) [16]	13-year-old boy	Benzathine penicillin	Intramuscular	Left gluteal region.	Multiple ecchymoses over the left gluteal region and back of the thigh, mild swelling of the left lower limb, and left foot drop, urinary and fecal incontinence	Oral prednisone 5 mg twice a day, tapered over the next two weeks, muscle-strengthening physiotherapy and intermittent electrical muscle stimulation therapy	Significant clinical recovery
Phiri et al. (2020) [17]	5-year-old boy	Benzathine penicillin	Intramuscular	Right lower limb	Painful, swollen right lower limb with dark discoloration extending from the toes to mid-thigh, with weak popliteal and dorsalis pedis pulses.	Imipenem 250 mg IV three times a day, metronidazole 200 mg IV, analgesia, and tramadol 25 mg intramuscular twice daily	The case was complicated with above-the-knee amputation of lower limb
Esme et al. (2021) [18]	34-year-old woman	Glatiramer acetate	Subcutaneous	Lower left abdomen	Painful, tender discoloration over her abdominal skin	Topical betamethasone valerate and mucopolysaccharide polysulfate cream twice daily	Complete regression of the lesion
Murdock et al. (2022) [19]	85-year-old woman	Fulvestrant	Intramuscular	Right gluteal region.	Necrotic lesion at the injection site	Multiple surgical debridement	The ulcer healed
Zargarbashi et al. (2023) [20]	6-year-old boy	Benzathine penicillin G	Intramuscular	Right lower limb	Pain, purple discoloration of the skin extended from the buttock to the toes. Edema leads to compartment syndrome of the buttock, thigh, and calf. After 30 h, no pulse and no sensory or motor responses were detectable in the right limb	Fasciotomy, multiple debridement	Below-knee amputation of the right lower limb
Ata et al. (2023) [5]	65-year-old woman	Diclofenac sodium	Intramuscular	Left gluteal region.	Pain and dark discoloration of the skin in the upper lateral quadrant of the left buttock	Surgical debridement, drain insertion and skin approximation with antibiotics	The wound healed completely with scarring.
Das et al. (2023) [21]	36-year-old man	Fluphenazine	Intramuscular	Right gluteal region	Dull pain, mild swelling, and necrotic ulceration over the injection site	Wound debridement was performed along with wide excision of the margins, and a prophylactic course of antibiotics was administered with 2 g of intravenous cefazolin for 5 days.	The wound has healed
Goli et al. (2023) [22]	32-year-old woman	Methocarbamol	Intramuscular	Left gluteal region	Severe pain, redness, and swelling involving her left buttock and the surrounding back area	The patient experienced rapid clinical deterioration	The patient died
Arunima et al. (2024) [23]	24-year-old man	Buprenorphine (tablet crushed in drinking water)	Intravenous	Upper lateral aspect of his right limb	Non-blanchable purpuric macules and patches in a retiform pattern over the sole of the right foot	Pentoxifylline 400 mg thrice daily and provided pain management	The patient could not be followed up
Bhuyyar et al. (2025) [24]	32-year-old woman	Diclofenac sodium	Intramuscular	Left gluteal region	Skin necrosis	Intravenous antibiotics and analgesics, followed by surgical debridement	The wound healed
Abtahi-Naeini et al. (2025) [25]	4-year-old girl	Triamcinolone acetonide	Intramuscular	Left gluteal region	Swelling and discoloration in the buttock area, and severe pain	Enoxaparin (1.5 mg/kg/dose twice daily), warm compresses, prophylactic antibiotics, and acetaminophen for analgesia	The patient healed
Cheng et al. (2025) [26]	39-year-old man	Penicillin G benzathine	Intramuscular	Left gluteal region	Painful rash on his left buttock	Corticosteroids, anticoagulation, fluid resuscitation, and alkalinizing the urine	The patient healed
Ramya et al. (2026) [27]	51-year-old man	Diclofenac sodium	Intramuscular	Gluteal region	Pain followed by dark discoloration and ultimately necrosis and ulceration	IV antibiotics, wound debridement	The patient is currently under follow-up.

## Data Availability

The original contributions presented in this study are included in the article. Further inquiries can be directed at the corresponding author.

## Abbreviations

CT	Computed tomography
MRI	Magnetic resonance imaging
STIs	Sexually transmitted infections

## References

[1] Gandino I.J., Majul B.J., Nuñez J., Ravagna M., Muñoz S.A., Gianni M. (2012). Síndrome de Nicolau posterior a la inyección de penicilina intramuscular. Actual SIDA.

[2] Peña Mira M.N., Valderrama Cuadros N., Lozano Ponce E.A. (2024). Síndrome de Nicolau: Afección rara y prevenible. Dermatol. Rev. Mex..

[3] Rodríguez Arias E.A., Sola M.F., de Arza Pochylak L., Molina M.F., Arana S., Alfaro C.T., Rodríguez Arias E.A., Sola M.F., de Arza Pochylak L., Molina M.F. (2023). Síndrome de Nicolau. Med. B. Aires.

[4] Nischal K., Basavaraj H., Swaroop M., Agrawal D., Sathyanarayana B., Umashankar N. (2009). Nicolau Syndrome: An Iatrogenic Cutaneous Necrosis. J. Cutan. Aesthetic Surg..

[5] Ata Y.M., Ahmed M.B., Al-Mohannadi F.S., Al-Jassim F.A., Shabbir A. (2023). Nicolau Syndrome Following Intramuscular Diclofenac Injection: A Case Report and Review of the Literature. J. Surg. Case Rep..

[6] Goudjo E.U.E.M., Metchihoungbe C.S., Mihluedo-Agbolan A.K., Houegban A.S.C.R., Miaffo O.E.D.D., Kante T.B., Sanni Y.S., Gnassingbe K. (2022). Nicolau Syndrome: A Series of Three Cases Following Drug’s Intramuscular Injection in Children. J. Pediatr. Surg. Case Rep..

[7] Taştekin F., Ersoy M., Aslan A., Meriç Özgenel Ş., Temel T., Özakyol A. (2017). Nicolau Syndrome. J. Turk Acad. Dermatol..

[8] Greaney J.L., Alexander L.M., Kenney W.L. (2015). Sympathetic Control of Reflex Cutaneous Vasoconstriction in Human Aging. J. Appl. Physiol..

[9] Izquierdo-Condoy J.S., Morales-Lapo E., Hidalgo M., Tello-De-la-Torre A., Ruiz-Sosa C., Guerrero-Castillo G.S., Sánchez Ordoñez D., Puglla A., Vasconez-Gonzáles J., Carrington S.J. (2023). Job Satisfaction and Self-Perceptions Among Ecuadorian Medical Doctors During Their Compulsory Rural Community Social Service: A Countrywide Cross-Sectional Analysis. J. Prim. Care Community Health.

[10] Ortiz-Prado E., Simbaña-Rivera K., Cevallos G., Gómez-Barreno L., Cevallos D., Lister A., Fernandez-Naranjo R., Ríos-Touma B., Vásconez-González J., Izquierdo-Condoy J.S. (2022). Waterborne Diseases and Ethnic-Related Disparities: A 10 Years Nationwide Mortality and Burden of Disease Analysis from Ecuador. Front. Public Health.

[11] Mojarrad P., Mollazadeh H., Barikbin B., Oghazian M.B. (2021). Nicolau syndrome: A review of case studies. Pharm. Sci..

[12] Navrazhina K., Cressey B.D., Wildman H.F. (2017). Nicolau Syndrome after Lumbar Puncture: A Case Report in a 22-Month-Old Girl. JAAD Case Rep..

[13] Kimbrough D.J., Newsome S.D. (2017). Case Report: Two Cases of Nicolau Syndrome Associated with Glatiramer Acetate. Int. J. MS Care.

[14] Ozlu E., Baykan A., Ertas R., Ulas Y., Ozyurt K., Avcı A., Baykan H. (2017). Case Report: Nicolau Syndrome Due to Etofenamate Injection. F1000Research.

[15] Gal S., Dart P.E., Movassaghi K. (2020). A Case Report of Nicolau Syndrome After Aesthetic Breast Surgery: A Review of the Literature and Introduction to a New Treatment Modality. Aesthetic Surg. J. Open Forum.

[16] Raju B., Ashraf O., Jumah F., Appaji Gowda N.M., Gupta G., Sun H., Nanda A. (2020). Nicolau Syndrome, Masquerader of Postinjection Sciatic Nerve Injury: Case Report and Review of Literature. World Neurosurg..

[17] Phiri W., Musonda M.S., Kyakilika K., Miyoba M.H., Malumani M. (2020). Nicolau Syndrome Following Intramuscular Benzathine Penicillin Injection: A Case Report. Pan Afr. Med. J..

[18] Esme P., Gahramanov I., Akıncıoglu E., Akoglu G. (2021). Nicolau Syndrome Following Subcutaneous Glatiramer Acetate Injection: A Case Report and Review of the Literature. Indian J. Pharmacol..

[19] Murdock J.L., Duco M.R., Sharma S.C., Reeves D.J. (2022). Embolia Cutis Medicamentosa (Nicolau Syndrome) Secondary to Intramuscular Fulvestrant Injection: A Case Report. J. Pharm. Pract..

[20] Zargarbashi R., Panjavi B., Keshavarz-Fathi M. (2023). Extensive Deep Tissue Involvement in Nicolau Syndrome and Below-Knee Amputation: A Case Report and Literature Review. Int. J. Low. Extrem. Wounds.

[21] Das S., Shet V., Jogarajah T., Ibrahim A., Reyes M., Fernandez Co E.M., Reddy B. (2023). Nicolau Syndrome Associated with Fluphenazine Depot: A Case Report. SAGE Open Med. Case Rep..

[22] Goli R., Faraji N., Janghiyamachi R., Talebiazar N. (2023). Nicolau Syndrome after Intramuscular Injection of Methocarbamol: A Rare Case Report. Toxicol. Rep..

[23] Arunima A., Singh S., Reddy A., Vinay K. (2024). Buprenorphine Induced Nicolau Syndrome: A Case Report. Indian J. Psychol. Med..

[24] Bhuyyar N., Khombare B., Panicker A., Teli S., Shalavadi M., Choudhari K. (2025). Nicolau Syndrome: Cutaneous Necrosis Following Diclofenac Intramuscular Injection. Georgian Med. News.

[25] Abtahi-Naeini B., Babaie S., Seyedyousefi S., Ahmadinia F. (2025). Nicolau Syndrome in a Pediatric Patient after Corticosteroid Injection: A Case Report and Review of the Literature. J. Med. Case Rep..

[26] Cheng Y., Mao Y., Wang J., Du X. (2025). Nicolau Syndrome Following Benzathine Benzylpenicillin Injection in a Patient with Syphilis and Type 2 Diabetes: A Case Report. Case Rep. Dermatol..

[27] Ramya A., Ambigai S., Swaminathan A. (2026). From Needle to Necrosis: A Case Report on Nicolau Syndrome. Cureus.

[28] Fekete G.L., Iantovics L.B., Fekete J.E., Fekete L. (2023). Embolia Cutis Medicamentosa (Nicolau Syndrome): Case Series. Front. Med..

[29] Al-Sheeb F., Al Mannai G., Tharupeedikayil S. (2022). Nicolau Syndrome after Endodontic Treatment: A Case Report. J. Endod..

[30] Martínez-Morán C., Espinosa-Lara P., Nájera L., Romero-Maté A., Córdoba S., Hernández-Núñez A., Borbujo J. (2011). Embolia cutis medicamentosa (síndrome de Nicolau) tras inyección de acetato de glatirámero. Actas Dermo-Sifiliogr..

[31] Hamilton B., Fowler P., Galloway H., Popovic N. (2008). Nicolau Syndrome in an Athlete Following Intra-Muscular Diclofenac Injection. Acta Orthop. Belg..

[32] Somasundaram A., Mathews J., Baby A., Chandrashekar L. (2025). Nicolau Syndrome Treated with Triple Therapy of Enoxaparin, Steroids, and Pentoxifylline and Monitored Using Infrared Thermography. Indian J. Dermatol. Venereol. Leprol..

[33] Harode S., Jain S.P., Ambulkar A. (2023). Nicolau Syndrome: A Dreaded Complication of Vitamin-12 Injection. Indian J. Drugs Dermatol..

[34] Segreto F., Tosi D., Marangi G.F., Gigliofiorito P., Pendolino A.L., Persichetti P. (2013). Nicolau’s Syndrome Complicated by Atypical Necrotizing Fasciitis. Arch. Plast. Surg..

[35] Hassan Y., Rasool H., Rather A.A., Wani M.H. (2023). The Nicolau Syndrome: A Case Report and a Review of the Literature. J. Surg. Trauma.

[36] Cárdenas E., Godos T., Lizarzaburu C., Benalcázar J., Lizarzaburu D. (2021). Tratamiento y manejo fibrinolítico en síndrome de Nicolau: Caso clínico. Metro Cienc..

[37] Sasmal P.K., Sahoo A., Singh P.K., Vs V. (2021). Nicolau Syndrome: An Unforeseen Yet Evadable Consequence of Intramuscular Injection. Surg. J..

[38] Enshaei A., Afshar A. (2016). Compartment Syndrome of the Calf Due to Nicolau Syndrome. Arch. Bone Jt. Surg..

[39] Pulido Pérez A., Parra Blanco V., Suárez Fernández R. (2013). Síndrome de Nicolau tras la administración de acetato de glatirámero. Neurología.

